# Mid-ventricular Takotsubo Cardiomyopathy With Coexisting Myocardial Bridge

**DOI:** 10.7759/cureus.54868

**Published:** 2024-02-25

**Authors:** Jasmine Dugal, Michael V DiCaro, Blaine Massey, Neelesh Gupta, Ahsan H Choudhury

**Affiliations:** 1 Internal Medicine, Kirk Kerkorian School of Medicine at University of Nevada, Las Vegas, Las Vegas, USA; 2 Cardiology, Kirk Kerkorian School of Medicine at University of Nevada, Las Vegas, Las Vegas, USA; 3 Cardiology, University Medical Center, Las Vegas, USA

**Keywords:** echocardiography, myocardial bridge, stress-related cardiomyopathy, cardiac cath, heart failure, internal medicine, cardiology

## Abstract

Typical takotsubo cardiomyopathy (TCM) is a reversible form of myocardial injury that presents with a characteristic ballooning abnormality of the left ventricular apex. Typical TCM has been associated with myocardial bridging; however, mid-ventricular variant TCM has not. We describe a rare case of mid-ventricular variant TCM with a coexisting left anterior descending artery myocardial bridge and discuss management strategies. Furthermore, we propose potential pathophysiological mechanisms that may contribute to the symptomatic presentation of both conditions as a manifestation of common etiological factors.

## Introduction

Stress-induced cardiomyopathy or takotsubo cardiomyopathy (TCM) is a reversible form of myocardial injury that presents with a characteristic ballooning abnormality of the left ventricle. Clinically, patients present with signs and symptoms mimicking acute coronary syndrome (ACS), including chest pain, diaphoresis, and anxiety. Classically, TCM is preceded by a significant physical or emotional stressor [1]. The typical variant of TCM affects the apex of the heart, causing apical ballooning in the left ventricle on imaging [1,2]. Atypical variants of TCM involve other parts of the left ventricle including the basal, focal, and mid-ventricular areas [1,3]. Per the International Takotsubo Registry, the mid-ventricular variant of TCM is only found in 14.6% of TCM cases, whereas the apical variant is seen in 82% of cases [4]. The basal and focal variants are seen in 4.5% and 1.1% of cases, respectively [4].

Myocardial bridging occurs when an epicardial coronary artery traverses the myocardium under a muscular bridge. This coronary anomaly most commonly occurs in the left anterior descending artery (LAD), resulting in the tunneled portion of the artery undergoing compression during ventricular systole. Myocardial bridging is often clinically silent [5]; however, like TCM, it can mimic ACS on presentation [6,7].

Myocardial bridging has been associated with apical TCM in previous studies, with incidences ranging from 11% to 76% [8-10]. To our knowledge, there are no documented reports of mid-ventricular variant TCM with coexisting LAD myocardial bridging. Here, we present a case of atypical mid-ventricular variant TCM causing circumferential mid-ventricular hypokinesia with basal and apical hypercontractility in the setting of LAD myocardial bridging.

## Case presentation

A 60-year-old woman with a past medical history of bipolar disorder, coronary artery disease (CAD), chronic obstructive pulmonary disease, hyperlipidemia, chronic kidney disease, anxiety, and obstructive sleep apnea was transferred to our emergency department from an outside facility with shortness of breath and pressure-like chest pain due to concern for non-ST elevation myocardial infarction (NSTEMI). The patient endorsed worsening non-exertional shortness of breath for four days, and intermittent non-exertional chest pain associated with palpitations and left arm pain for the past eight months. Over the course of the prior week, the chest pain had worsened, which prompted her to seek treatment.

**Figure 1:**
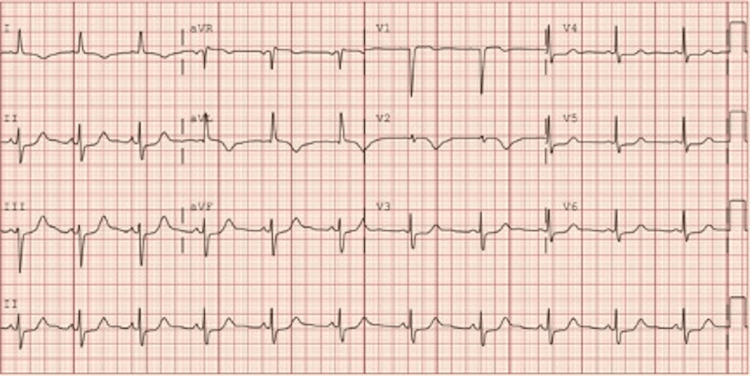
Initial EKG showing normal sinus rhythm with left axis deviation, T wave inversions in leads I, V2, and avL, along with ST-segment depression in leads II, III, avF, and V3-V6. aVL: augmented vector left; aVF: augmented vector foot.

Upon initial evaluation, the patient was found to have a high-sensitivity troponin of 243 ng/L. A computed tomography (CT) scan of the chest was unremarkable. She received aspirin, morphine, and heparin bolus before being transferred to our facility. The physical examination was unremarkable with the exception of bilateral expiratory wheezing. Electrocardiogram (EKG; Figure 1) showed sinus rhythm with left axis deviation, T wave inversions in leads I, V2, and augmented vector left (avL), along with ST-segment depression in leads II, III, augmented vector foot (avF), and V3-V6. The patient was admitted to our hospital and re-started on a heparin infusion with the intention to perform an urgent coronary angiography.

**Figure 2:**
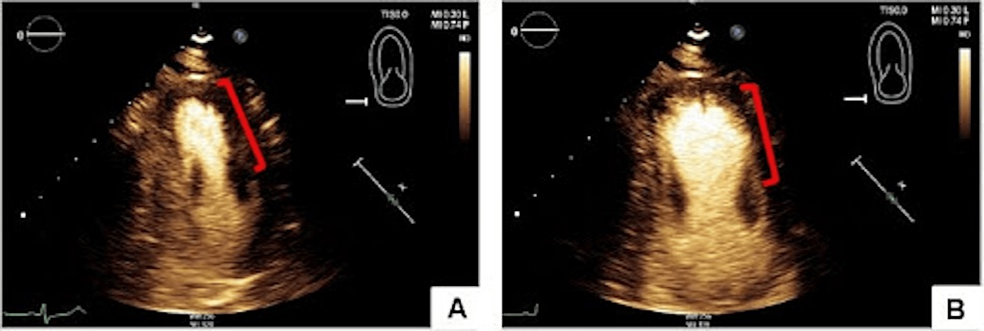
Echocardiography in apical view with perflutren lipid microsphere contrast demonstrating LV chamber in systole (A) and diastole (B). Note the LV ballooning and anterior wall hypokinesis in diastole and systole indicated by the red brackets. LV: left ventricle.

**Figure 3:**
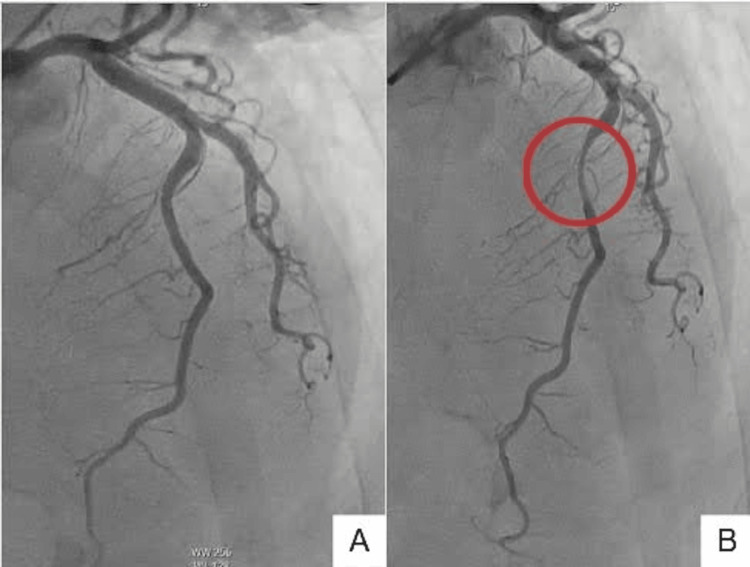
Coronary angiogram in RAO cranial view showing LAD myocardial bridge. The difference in diameter is noted in the myocardial bridging of LAD in diastole (A) versus systole (B) (red circle). RAO: right anterior oblique; LAD: left anterior descending.

**Figure 4:**
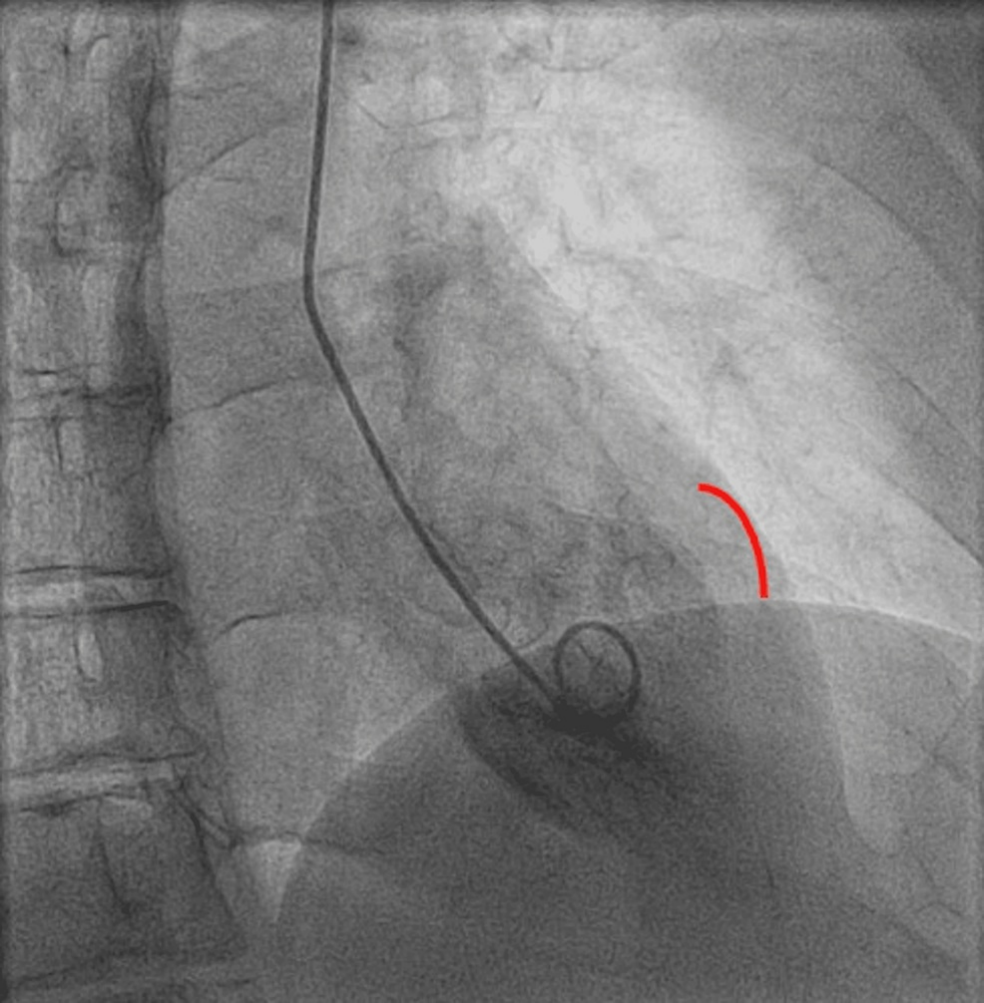
Left ventriculogram showing akinetic/hypokinetic mid-anterior wall (red line) in comparison to the normal basal and apical portion, which shows intact contractility.

Repeat high-sensitivity troponin decreased to 140 ng/L. Other relevant labs included a brain natriuretic peptide (BNP) of 114 pg/mL, activated partial thromboplastin time (apTT) of 56 seconds, glomerular filtration rate (GFR) of 47 ml/min, total cholesterol of 233 mg/dL, triglycerides of 241 mg/dL, low-density lipoprotein (LDL) of 124 mg/dL, and high-density lipoprotein (HDL) of 46 mg/dL. Transthoracic echocardiogram showed mid-ventricular anterior wall hypokinesis with reduced left ventricular ejection fraction of 40-45% (Figure 2). The patient was taken for coronary angiography, which revealed a LAD myocardial bridge and non-obstructive CAD (Figure 3). The left ventriculogram showed a hypokinetic mid-anterior wall, suggestive of mid-ventricular variant stress-induced cardiomyopathy (Figure 4).

The patient was prescribed diltiazem for the myocardial bridge and was instructed to follow up with her primary care physician as well as outpatient cardiology within the next two weeks. Per chart review of follow-up records, the patient's symptoms have been well-controlled since discharge.

## Discussion

Several studies have shown a possible association between apical TCM and myocardial bridging; however, pathophysiologic mechanisms implicating the two conditions are not fully understood. TCM occurs due to a combination of acute physiologic changes manifesting as a result of emotional or physiological stress, including coronary artery spasm, microvascular dysfunction, and catecholamine surge leading to catecholamine-induced myocardial stunning [2]. Similarly, it has been proposed that symptomatic myocardial bridging is seen more frequently in patients experiencing increased cardiac stress secondary to elevated sympathetic activity [11-13]. Increased sympathetic activity can lead to increased inotropy and chronotropy via stimulation of B1 adrenergic receptors, and increased vasospasm via A1 adrenergic receptor-mediated vasoconstriction. Studies that combined intracoronary Doppler flow and pressure measurements with coronary angiograms have suggested a decrease in diastolic vessel diameter and decreased coronary blood flow in symptomatic myocardial bridging [5]. This mechanism suggests that tachycardia or greater contractility, which results in decreased diastolic coronary artery filling time, may result in myocardial ischemia in the distribution of the bridged vessels. Therefore, increased catecholaminergic activity during situations of high physical or emotional stress may precipitate both TCM and symptomatic bridging. It is also possible that the myocardial bridge is merely an anatomical variant and may not be associated with TCM [12]. Furthermore, there has not yet been an association between mid-ventricular TCM and myocardial bridging, and it is currently unknown whether the location of the ventricular wall hypokinesis is influenced by the bridge, or vice versa.

TCM and myocardial bridging, whether co-existent or not, clinically present similarly to classic ACS. ACS requires prompt recognition and intervention to prevent long-term structural and functional heart debilitation. In the event of an ST-segment elevation myocardial infarction, timely angiography with percutaneous coronary intervention (PCI) is crucial. Patients are then started on optimal medical therapy for CAD, which includes dual antiplatelet therapy with a P2Y12 receptor antagonist such as clopidogrel or Brilinta and aspirin. It also includes atorvastatin and a beta blocker in most cases. In NSTEMI and unstable angina, patients are typically started on anti-coagulation therapy, such as intravenous heparin, and are taken for angiography within 24 hours. It is important to note the difference in the management of these conditions in contrast to the management of ACS. Treatment of TCM, a transient disorder, is typically supportive management, which includes the elimination of the physical and emotional stressors that can help alleviate symptoms [1]. It has been hypothesized that long-term beta-blocker therapy may reduce the likelihood of recurrence and can improve symptoms of TCM [4]. Other management of TCM includes preventing the development of acute decompensated congestive heart failure, which can progress to cardiogenic shock. Additionally, mid-ventricular TCM increases the risk of development of pleural or pericardial effusions and intraventricular thrombus in severe left ventricular dysfunction [4]. These potential complications warrant close clinical monitoring before discharge.

Symptomatic myocardial bridging may require medical or even surgical therapy for both short and long-term treatment. Calcium channel blockers have proven to be beneficial in myocardial bridging if vasospasm is present [5,14]. Beta-blockers are useful in relieving hemodynamic stress on the heart by reducing chronotropy and inotropy, and increasing diastolic filling time for coronary arteries [5]. In the 1990s, PCI was often used to resolve hemodynamic abnormalities and symptoms caused by a myocardial bridge [5,14]. With PCI therapy, complications like target lesion revascularization, stent fracture, stent thrombosis, and coronary perforation resulted [5]. Now, more common surgical therapies for myocardial bridge include coronary artery bypass grafting (CABG) and myotomy. CABG therapy has demonstrated graft success in long and deep myocardial bridging [5]. For shorter or more superficial myocardial bridges, surgical myotomy has demonstrated increased coronary blood flow and elimination of symptoms [5]. Medical therapy with calcium channel blockers remains the first-line therapeutic strategy. Surgical therapy is rarely pursued in the modern era of practice.

The possible association between TCM and myocardial bridging may be a result of manifestations related to high-stress environments. Increased sympathetic activity during periods of intense physical or emotional stress may result in enhanced inotropic and chronotropic activity, which results in shortened diastole, aggravating systolic compression of an otherwise asymptomatic myocardial bridge [15]. It is unclear if the symptomatic bridge may be a contributing factor in the development of TCM. Despite their possible overlapping etiologies, coexisting or stand-alone TCM and myocardial bridging may present similarly to ACS. It becomes important to keep a broad differential and distinguish between these pathologies when patients present with anginal chest pain due to differences in management.

## Conclusions

This case adds to the growing body of literature that may support an association between myocardial bridging and TCM. The exact nature of this association is hypothesized but not fully understood and may be due to common etiologic factors. Furthermore, this association was formerly only described with apical TCM. This case presents a rare and intriguing clinical scenario involving mid-ventricular variant TCM with a coexisting LAD myocardial bridge.
